# Refinement of the motorised laminectomy-assisted rat spinal cord injury model by analgesic treatment

**DOI:** 10.1371/journal.pone.0294720

**Published:** 2024-01-16

**Authors:** Harikrishnan Vijayakumar Sreelatha, Hamza Palekkodan, Ansar Fasaludeen, Lissy K. Krishnan, Klas S. P. Abelson

**Affiliations:** 1 Department of Applied Biology, Division of Laboratory Animal Science, Biomedical Technology Wing, Sree Chitra Tirunal Institute for Medical Sciences and Technology, Thiruvananthapuram, Kerala, India; 2 Department of Experimental Medicine, Faculty of Health and Medical Sciences, University of Copenhagen, Copenhagen, Denmark; 3 Department of Veterinary Pathology, College of Veterinary and Animal Sciences, Pookot, Wayanad, Kerala, India; 4 Biological Research and Innovation Wing, Dr. Moopen’s Medical College, Wayanad, Kerala, India; Belgrade University Faculty of Medicine, SERBIA

## Abstract

Usage and reporting of analgesia in animal models of spinal cord injury (SCI) have been sparse and requires proper attention. The majority of experimental SCI research uses rats as an animal model. This study aimed to probe into the effects of some commonly used regimens with NSAIDs and opioids on well-being of the rats as well as on the functional outcome of the model. This eight-week study used forty-two female Wistar rats (Crl: WI), randomly and equally divided into 6 treatment groups, viz. I) tramadol (5mg/kg) and buprenorphine (0.05mg/kg); II) carprofen (5mg/kg) and buprenorphine (0.05mg/kg); III) carprofen (5mg/kg); IV) meloxicam (1mg/kg) and buprenorphine (0.05mg/kg); V) meloxicam (1mg/kg); and VI) no analgesia (0.5 ml sterile saline). Buprenorphine was administered twice daily whereas other treatments were given once daily for five days post-operatively. Injections were given subcutaneously. All animals underwent dental burr-assisted laminectomy at the T10-T11 vertebra level. A custom-built calibrated spring-loaded 200 kilodynes force deliverer was used to induce severe SCI. Weekly body weight scores, Rat Grimace Scale (RGS), and dark-phase home cage activity were used as markers for well-being. Weekly Basso Beattie and Bresnahan (BBB) scores served as markers for functionality together with Novel Object Recognition test (NOR) at week 8 and terminal histopathology using area of vacuolisation and live neuronal count from the ventral horns of spinal cord. It was concluded that the usage of analgesia improved animal wellbeing while having no effects on the functional aspects of the animal model in comparison to the animals that received no analgesics.

## Introduction

In the present research community, animal welfare bodies, organizations, animal ethical committees, regulators and funding agencies are increasingly requiring analgesia to be the part of protocols under high-severity classification with long-lasting pain and distress in experimental animals. However, such requirements may take no consideration neither to the actual beneficial effects for the animals, nor to any possible interference with functional outcomes of the study. Hence, a thorough investigation on dosage and combinations of analgesic drugs to ensure animal well-being without intervening with the functional outcome measures should be performed for all experimental model, which was also the overall objective of this study. In animal experimentation involving surgical procedures, where the animals are obviously subjected to severe pain as a result of the experimental intervention, a considerable percentage (71%) has no mention about analgesic treatment [1–3]. It is important to note that reproducibility crises in research can only be overcome with proper reporting [4]. It was recently established using a systematic review that anesthesia and analgesia was not reported in 87.8% of the cases in the year 2009 and 74.8% of the cases in 2019 in rats and mice that underwent craniotomy [5]. In a systematic review that was published in 2016 to assess the usage of analgesia in laboratory animals, it was found that only 30% mice in spinal surgeries and 10% rats in neuropathy models received analgesics postoperatively [1, 3]. It has been proven that providing pain relief can benefit the wellbeing of various experimental animal models also the outcome of research in many cases [6–8]. Reviewers and funding agencies are moving in a direction where questions are raised in the cases where analgesia is not provided to experimental animals, since refraining from analgesic treatment can make the scenario totally different from human cases and thus potentially affect extrapolation [3]. Based on this, more work is required to elucidate the effect of analgesics on the outcome of the experimental read-out in various animal models. It shall also be noted that spinal cord injury (SCI) research uses multiple species widely and hence, it is important to see the effects of analgesia on animal welfare in all these species.

Animal models have unique features depending on the purpose, and the interaction of analgesic drugs with experimental read-outs may be different. Interactions may in turn be dependent on sex, strain and types of analgesia used [9–11]. There is impinging evidence that, sex differences in behavioural responses to opioids exists, primarily in rats [12]. However, differences in response to analgesia may also be related to strain. In a study where male Lewis rats were treated with buprenorphine without having any impact on the outcome in the expression of rheumatic arthritis [9], whereas treated female Lew/SSN rats were affected with rheumatic arthritis [10]. Hence, this difference could be both due to sex and to strain related genetic differences, as Lew and Lew/SSN are different lines of the same stock [13]. Likewise, the experimental method for inducing arthritis may have an impact as well, since different methods were applied in the two studies referred to [9, 10]. Acetaminophen [14] and morphine [9] were shown to exhibit anti-arthritic effects, whereas gabapentin did not interfere with the progression of arthritis [11]. Middle cerebral artery occlusion stroke model in mice is a widely used model and one study demonstrated that the infarct size was significantly reduced by meloxicam whereas buprenorphine did not affect the infarct post-surgically.

The present study of ours is an attempt to investigate the effects of different types of analgesic drugs on the levels of well-being of the animals and to compare their effects on the functional outcome of contusion-induced SCI in female Wistar rats. The analgesic drugs and combinations were chosen from previous reports that aimed to minimize pain and suffering in various rat models [2, 15–17]. The study also employed the previously validated protocol of mechanising the process of creating laminectomy using a motorised dental burr.

Assessment and interpretation of pain is a major part in applying an appropriate analgesic regimen to experimental rodents [18], and the study was therefore designed with an array of tests of relevant behavioural parameters to generally assess health status and progress of post-operative recovery of animals, clinical signs and body weight changes. To assess the functionality of the model, the Basso Beattie Bresnahan (BBB) score in open field was recorded, to assess the level of paraplegia as described previously by Basso *et al* [19]. Since SCI animal models are also used as models of pain [20, 21] and memory loss [22–24], mechanical allodynia was tested using von Frey filaments, and a modified novel object recognition test (NOR) optimized for paraplegic rats was employed [25, 26].

It was hypothesised that analgesia would have a positive impact on animal well-being, and that multimodal analgesia would be superior to NSAIDs being used independently. It was also hypothesised that analgesic treatment would not cause any interaction in the development and progression of the BBB score, on behavioural parameters or on lesions in the spinal cord necessary to generate a valid animal model of SCI.

## Materials and methods

### Animal care and ethical approval

The Institutional Animal Ethical Committee of Sree Chitra Tirunal Institute for Medical Sciences and Technology sanctioned this project (B Form approval number SCT/IAEC-367/JULY/2020/106, dated 27^th^ July 2020), which is as per the guidelines issued under the statutory framework of The Committee for the Control and Supervision of Experiments on Animals (CCSEA).

The animals, female (Crl: WI) rats, 9–12 weeks of age and body weight of 240–280 gram upon arrival, were procured from Charles River Laboratories through Hylasco bio, India Limited, Bangalore and were housed under controlled environmental conditions for the study. Animals had a health status that was in compliance with the FELASA guidelines [27]. ARRIVE checklist was used to maintain compliance to the guidelines [28] for the study. Food (Safe rodent diet, D131, Augy, France) and water was given *ad libitum*, in individually ventilated rat cages made of polysulfone with 800 cm^2^ floor area and 18.5 cm height (Citizen Industries Limited, Ahmedabad, India). The animal holding rooms were maintained at a temperature of 22 ± 2°C, 30–70% relative humidity, 15 air changes per hour, and light levels not exceeding 325 Lux at one meter height from the floor. The facility ensured that no prolonged noises above 85dB prevailed throughout the study. Corncob was autoclaved and was used as bedding material (Sparcobb, India) and paper for net building (Enviro Dri, Shepherd, Cleveland, Ohio, USA) was provided throughout the study with cage changes done once every 5 days. Autoclaved wooden chewing blocks (Kansara Scientific, India) was provided to all the rats throughout the study. Animals were housed in pairs on arrival, and were singly housed one week before the surgery to get acclimatized. The animals were expected to be very stressed during the first week after surgery, when the animals are totally paralytic, requiring more floor space to move around. Therefore, single-housing is applied as a standard for the first week, since they appear less stressed this way. This phase is the most critical period and once they tide over this phase, they were pair-housed. To avoid additional stress from changing environment from pair- to single-housing immediately after surgery, the animals were acclimatised to single housing for seven days prior to surgery. Handling and conditioning of the rats were done twice a week. To avoid cage effect, after randomisation, animals from different groups were mixed to be housed in pairs in each cage. For all the experiments, each single animal was considered as the experimental unit. Experimental procedures were done between 9.00 am and 2.00 pm and the dark-phase activity video recordings were done between 7.00 pm-11.00 pm.

### Study design

The sample size was based on previous studies [25, 29] and set to seven animals per group. Randomization was done using (https://www.randomizer.org/). The forty-two rats were uniquely marked for identification, pair-housed, and assigned to undergo motorised dental burr assisted laminectomy followed by spinal cord injury and to receive various analgesic treatments thereafter. The analgesic used were tramadol (Supridol, Neon Laboratories, Mumbai, India), buprenorphine (Bupregesic, Neon Laboratories, Palghar, India), carprofen (Carpade, Carus Labs, Karnal, India) and meloxicam (Melonex, Intas Laboratories, Ahmedabad, India). Application of the analgesics in the different treatment groups are shown in Table 1. All substances were delivered subcutaneously during five days. After grouping into six groups viz Group I to VI, animals were offered generic numbers and after the statistical analysis, the investigators and histopathologists were provided with their identity to correlate with the allotted treatment group.

**Table 1:** Application of the analgesics in the different treatment groups.

Groups	Group I	Group II	Group III	Group IV	Group V	Group VI
Analgesic regimen	Tramadol 5mg/kg + buprenorphine 0.05mg/kg Buprenorphine twice daily; tramadol once daily	Carprofen 5mg/kg + buprenorphine 0.05mg/kg Buprenorphine twice daily; carprofen once daily	Carprofen 5mg/kg once daily	Meloxicam 1mg/kg + buprenorphine 0.05mg/kg Buprenorphine twice daily; meloxicam once daily	Meloxicam 1mg/kg once daily	No analgesia control–sterile saline 0.5 ml once daily

### Anesthesia and surgery

Induction of anesthesia was done using 5mg/kg body weight xylazine (Xylaxin, Indian Immunologicals, Hyderabad, India) and 80mg/kg body weight ketamine (Aneket, Neon Laboratories limited, Thane, India). Drugs were injected intraperitoneally after mixing the drugs in one syringe. Anaesthesia was maintained using 2% Isofluorane (Forane, Abbott India Limited, Mumbai, India) administered via a face mask from a precision vaporizer (E-Z system corporation, Palmer, PA, USA). Eye protection was achieved intra-operatively by applying eye ointment immediately after the induction of anesthesia (Neosporin, GlaxoSmithKline pharmaceuticals Ltd. Mumbai, India). Analgesia was given as per the study design, and was injected immediately after the induction of anesthesia. After the pedal pinch reflex was negative, the dorsum of the rats was clipped and povidone iodine wipe was applied. The animals were draped with sterile window drapes and under strict asepsis, a 2.5 cm skin incision was made, the paraspinous muscles were separated by blunt dissection and–using a micro rongeur–the dorsal spine was resected. All the animals were operated at the level of the T10-T11 vertebra after radiographic confirmation of the site. The laminectomies were performed using a motorised dental burr as previously described in detail by Harikrishnan *et al* [25, 29]. The dorsal vertebral walls on both the sides were drilled using the burr (Carbide burrs, SSWHP-559, NJ, USA) equipped with a micromotor (Marathon-4, max RPM-35000, SDE-H37LI, Saeyang Microtech, Korea). The burr motor was controlled with a foot switch. Sterile saline was continuously instilled to the area to prevent thermal damage of underlying spinal tissue. The drilling was done in such a way to make channels that extended throughout the entire length of the vertebra on both the sides, which loosens the bony connections and attachments for a less forceful and atraumatic removal. After the bones were loose the pieces were removed smoothly using a jeweller’s forceps, to visualize the spinal cord. Using a 2.5mm impounder tip, 200 kilodynes impact was delivered to produce a severe contusion injury, with a custom-fabricated spring-loaded force deliverer [25, 29]. Immediate reflexes manifested as sudden extension of hindlimbs, urination and observation of hematoma at the site were considered signs of desired spinal cord injury. Muscles were sutured using 3–0 braided polyglactin sutures with half circle round bodied needle (Lotus, Dehradun, India) in a continuous lockstitch pattern and the skin was closed using interrupted sutures with 3–0 braided polyglactin cutting edged half circle needle. Povidone iodine ointment was applied over the skin wound and 5 ml of sterile isotonic saline was subcutaneously injected at the dorsal neck region as fluid therapy to improve post-op recovery. The animals were placed in a warm recovery area and were transferred back to home cages as soon as the animal’s righting reflex was positive. All animals recovered as expected, without any urinary infections or weight loss of 20%. Hence, all animals entering the study completed the term of 56 days uneventfully. The duration of surgery, from the time of incision to the completion of last skin suture, did not differ between groups. The mean duration of surgery in minutes were 21.57±1.9 for the tramadol and buprenorphine group, 22.14±1.5 for carprofen and buprenorphine, 22.43± 1.6 for carprofen, 20.28± 1.8 for meloxicam and buprenorphine, 22.57± 0.7 for meloxicam, and 21.43±1.7 for the no analgesia group.

### Post-operative care and clinical observations

Ceftriaxone (Intas pharmaceuticals, Ahmedabad, India) injection was given once daily at a dose of 15mg/kg to all rats for five days subcutaneously, to decrease complications from urine retention. A complication associated with the model is retention of urine, lasting for two weeks. Therefore, the animals were restrained twice daily for examination and assisted in micturition with the thumb gently pressed over the lower abdomen. On the seventh day, wounds were examined to observe proper healing and residual sutures, if any, were removed. From this stage, the animals were pair-housed. Throughout the study, animals were provided with two wheat biscuits and five grams of sprouted Bengal gram. This was to provide the animals with a softer option of food in comparison to the pelleted food to promote eating and better digestion, and as a food treat to provide them with enrichment during the recovery phase from the high severity procedure. Humane endpoints had been pre-determined, and if animals losing 20% of their basal body weight on Day 0 were to be euthanised. Further, it was pre-determined that any animal showing persistence of pus in urine and self-mutilation to a considerable level also were to be immediately euthanised and excluded from the study. After two weeks, it was made mandatory that the animals be handled once daily and clinical observations drawn to ensure their well-being.

### Data collection

#### Body weight

Body weight recordings were made in all groups on Day 0 (the day of surgery), and on Day 1, 7, 14, 21, 28 and 56.

#### Rat grimace scale (RGS) scoring

Rat Grimace Scale (RGS) scoring was used to assess spontaneously occurring pain, and hence applied to monitor the post procedural animal well-being. The RGS recording box was an acrylic box of 25x12x12 cms (E-Z system corporation, Palmer, PA) with longitudinal sides made fully covered using opaque black plastic material. The animals were acclimatized to the recording box by being placed there twice daily for five to ten minutes, during seven days before the study was initiated. Boxes were filled with a layer of sterile corncob bedding during acclimatization and testing. Two video cameras (Sony HDR-CX405, Tokyo, Japan) were placed at the transparent ends of the assessment box. The top side was covered with an SS 304 grill for aeration and video recording was performed for 5 minutes. Immediately after the video recordings, the animals were transferred back to their home cages. One clear still image from either one of the cameras, each minute was extracted (based upon availability and clarity) and was analysed for the four action units viz orbital tightening, nose/cheek flattening, ear changes and whisker changes. Each action unit is scored in three levels–zero (no pain), one (moderate pain) and two (obvious pain) [30]. RGS scores from each action unit were averaged and the total score was noted for each animal.

#### Dark phase home cage activity

Recordings of animal activity in their home cages during the dark phase was made on Days 1, 7, 14, 21, 28 and 56. Activity was recorded for 5 minutes using a night vision camera fixed on a tripod inside the animal rooms as described previously [25]. Time in seconds of animal activity was compared between the groups. Activity was defined as eating, drinking, moving around, playing or interacting with the cage mate, rearing and grooming. Stereotypic activity monitoring was also screened which was defined as repetitive meaningless activities without the motive to perform any goal or biological function. On Day 1, the animals were individually housed. Recordings from Day 7 are from animals that were housed in pairs.

#### Basso beattie bresnahan (BBB) scoring

Motor functionality of hind limbs was done using BBB scoring on Day 1, 7, 14, 21, 28 and 56. The score is a non-linear scale where normally ambulating animals will have a score of 21, which is the maximum score that can be assigned, while animals with a severe spinal cord injury will have a score of zero, which indicates no observable movement at all. Any animals that did not develop the BBB score 0 on Day 1 was decided to be excluded from the study. The inclusion criteria of zero BBB scores during the first post-operative day was met by all animals subjected to SCI.

#### Novel object recognition (NOR) test

The set up to conduct the NOR test was designed specifically for posteriorly paralysed animals as described previously [25]. Baseline data was obtained prior to surgery to analyse whether the memory of the animals was intact. Initially, for the familiarizing phase, animals were exposed to two similar objects (two cubes with 5 cm length for a side or two spheres with 8 cm diameter of same colours) for 5 minutes. After a 5-minute delay and a 24 hours delay, a novel object was introduced by replacing one familiar object whereas the other familiar object is retained and the animals were let into the set up for 5 minutes. Exploration of the object was defined as sniffing, biting, touching with fore paws, leaning or rearing onto the object and observing from very close vicinity. Video recordings were made of all the sessions. In between animals, the objects were wiped using 70% ethanol. Discrimination Index (DI) was measured (DI = T_N_-T_F_/T_N_+T_F_, where T_N_ is the time spent with the novel object and T_F_ is the time spent with the familiar object) and were compared between the groups. The test was conducted on Day 56 at 5 minutes and 24 hours delay again to assess the memory loss owing to SCI.

#### von Frey test

von Frey test was performed by stimulating the dorsal part of the hind paw, as previously described [26], since all the animals studied manifested posterior paralysis owing to SCI. Stimulation was made between first and second metatarsal on the dorsal side, immediately above the joint, with 4 g von Frey Filament (VFF) as the minimum and 60 g VFF was the maximum used. Out of the three trials each time, a minimum of two responses was considered as positive response. In the case of a positive response, the subsequent thinner filament was applied, and if a negative response was seen, so the next thicker filament was applied. The lowest filament evoking response was considered the final score and the test was stopped at that point.

#### Euthanasia

At the end of the study, the animals were euthanised by carbon dioxide inhalation in a chamber and samples were collected for histopathology.

#### Histopathology

Histopathological analysis was done on 5 μm sections from the lesion epicentre at the spinal cord ventral horn region on both sides. Sections were stained using hematoxylin eosin for assessing the total vacuole area and using Cresyl violet (Nissl’s) stains to assess the live neuron count in the section [31, 32]. Area of intact spinal cord from the T10-T11 region of a rat of same age, weight, sex and strain that did not undergo SCI was used as a control for comparison. Formalin fixation in 10% neutral buffered formalin and decalcification were followed as described previously [25]. The vacuole area was analysed using ImageJ software (U. S. National Institutes of Health, Bethesda, Maryland, USA) and was compared between groups. Neuronal count was manually done by two experienced and trained histopathologists, blinded to the treatments.

### Statistical analysis

Statistical analysis was performed using GraphPad Prism for Windows version 9.4.1, 2022 (GraphPad Software, San Diego, California USA www.graphpad.com). All data is expressed as mean value for each group, and standard deviation (Mean±SD). Data was tested for normality using the Shapiro-Wilk test and normally distributed data was analysed with one-way analysis of variance (ANOVA). Data that did not follow normal distribution were analysed using Kruskal-Wallis test. Tukey’s multiple comparison test was used to perform post-hoc analysis to find differences between groups, following the ANOVA. Dunn’s post-hoc multiple comparison test was used following the Kruskal-Wallis test, for between-group comparisons. In all the cases, P < 0.05 was considered statistically significant. Data is made available in public repository https://doi.org/10.6084/m9.figshare.23522880.

## Results

### Body weight

**Fig 1:**
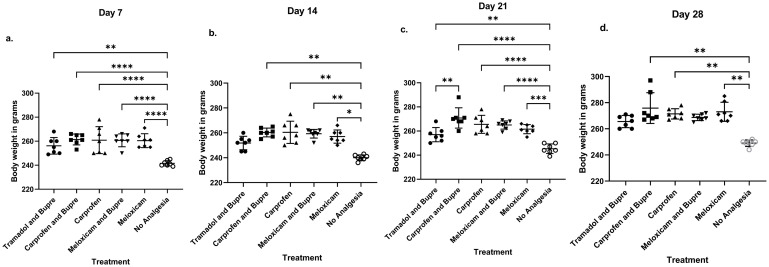
Body weight comparison on days 7(a), 14 (b), 21 (c) and 28 (d) showed differences between groups. * = P < 0.05, ** = P < 0.01, *** = P < 0.001, **** P <0.0001.

The animals did not differ in the baseline body weight on Day 0, measured before induction of anaesthesia. The mean initial body weight in grams on the day of surgery was 274.7±1.9 for the tramadol and buprenorphine group, 275.86±2.7 for carprofen and buprenorphine, 275± 1.6 for carprofen, 275.7± 2.9 for meloxicam and buprenorphine, 276.71± 2.1 for meloxicam, and 275.7±2.9 for the no analgesia group. The groups differed significantly in body weight on Day 7 (F (5,36) = 9.56, P< 0.0001), Day 14 (Kruskal-Wallis statistic = 23.28), Day 21 (F (5,36 = 16.33, P < 0.0001) and Day 28 (Kruskal- Wallis statistic = 22.04) (Fig 1). The groups did not differ on the first post-operative day, *Day 1*. On *Day 7*, post hoc test revealed that the no-analgesia group had significantly lower body weights in comparison with the tramadol and buprenorphine group (P = 0.0027), carprofen and buprenorphine (P< 0.0001), carprofen (P< 0.0001), meloxicam and buprenorphine (P < 0.0001) and meloxicam (P < 0.0001) (Fig 1a). On *Day 14*, the groups carprofen and buprenorphine (P = 0.001), carprofen (p = 0.0051), meloxicam and buprenorphine (P = 0.003) and meloxicam (P = 0.046) showed significantly higher body weight compared to the rats in the no analgesia group (Fig 1b). Post-hoc comparisons showed that tramadol and buprenorphine group had lower body weight with respect to carprofen and buprenorphine group on *Day 21* (P = 0.0011). Moreover, Day 21 also showed that the tramadol and buprenorphine group (P = 0.0074), carprofen and buprenorphine (P< 0.0001), carprofen (P< 0.0001), meloxicam and buprenorphine (P< 0.0001), and meloxicam (P = 0.0001) groups showed more body weights in comparison to the no-analgesia group (Fig 1c). Carprofen and buprenorphine (P = 0.0021), carprofen (P = 0.003) and meloxicam (P = 0.0035) had higher body weight in comparison to the no analgesia group on *Day 28* (Fig 1d). The groups did not differ on the Day 56 post-operatively.

### Rat Grimace Scale (RGS) scoring

**Fig 2:**
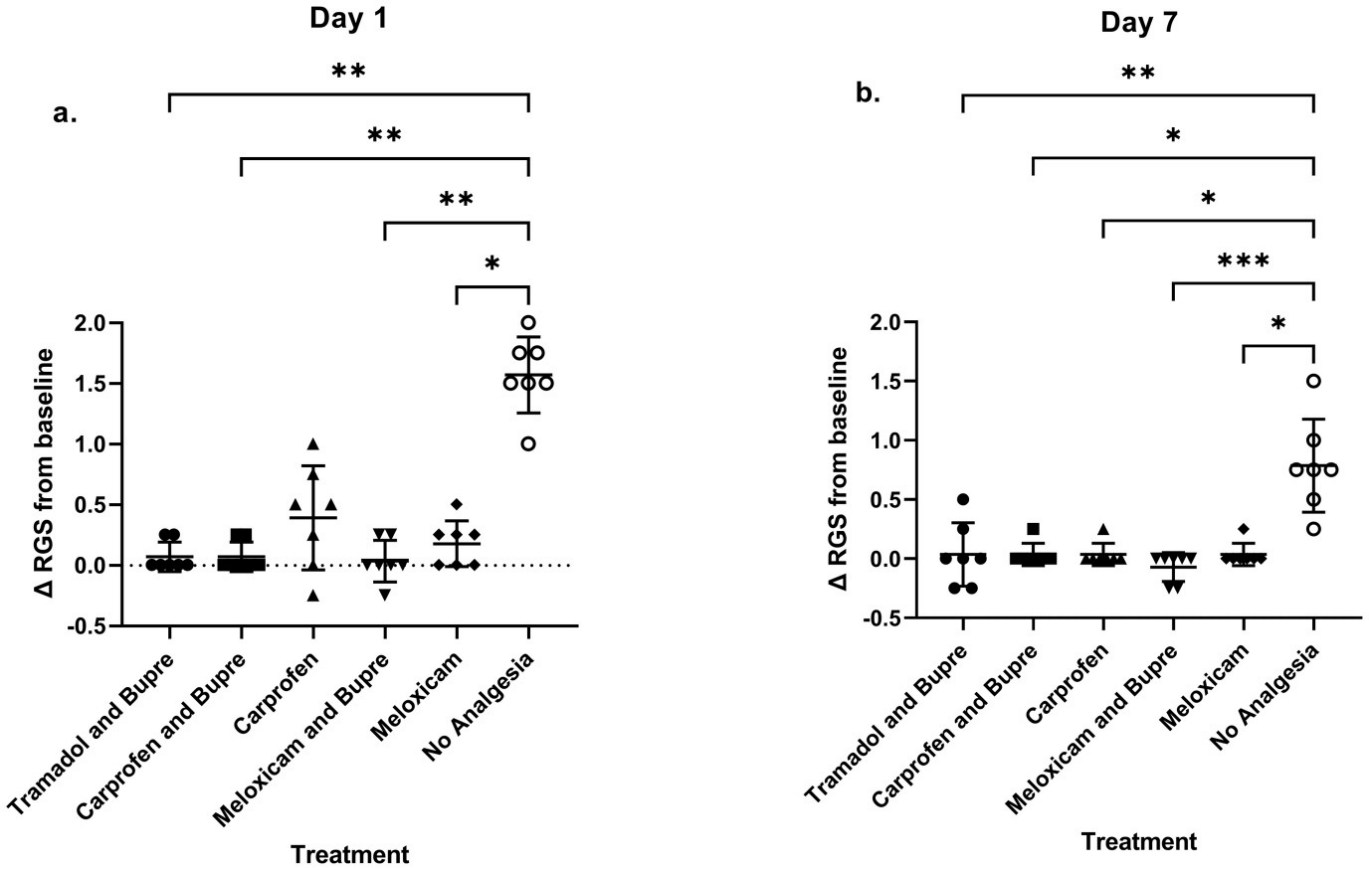
Rat Grimace Scale showed changes between groups with respect to the baseline on days 7 (a) and 14 (b). * = P < 0.05, ** = P < 0.01, *** = P < 0.001.

The RGS scoring clearly revealed the presence of spontaneously occurring pain that could be categorized from moderate to obviously present in the no-analgesia group on Day 1 (Kruskal- Wallis statistic = 22.64) and Day 7 (Kruskal- Wallis statistic = 23.16) (Fig 2). In contrast, spontaneously occurring pain was not detectable in any of the groups that received a treatment with analgesia, as no difference form baseline scores were registered.

On *Day 1*, Dunn’s post-hoc test showed that the no-analgesia group had a higher RGS scores in comparison to the tramadol and buprenorphine group (P = 0.0026), carprofen and buprenorphine (P = 0.0026), meloxicam and buprenorphine (P = 0.001) and meloxicam group (P = 0.0459) (Fig 2a).

On *Day 7*, post-hoc analysis showed that the no-analgesia group exhibited higher RGS scores in comparison to the tramadol and buprenorphine (P = 0.0055), carprofen and buprenorphine (P = 0.0153), carprofen (P = 0.0153), meloxicam and buprenorphine (P = 0.0001) and meloxicam (P = 0.0153) groups. (Fig 2b). Groups did not differ in RGS scores on any other days.

### Dark phase home cage activity

**Fig 3:**
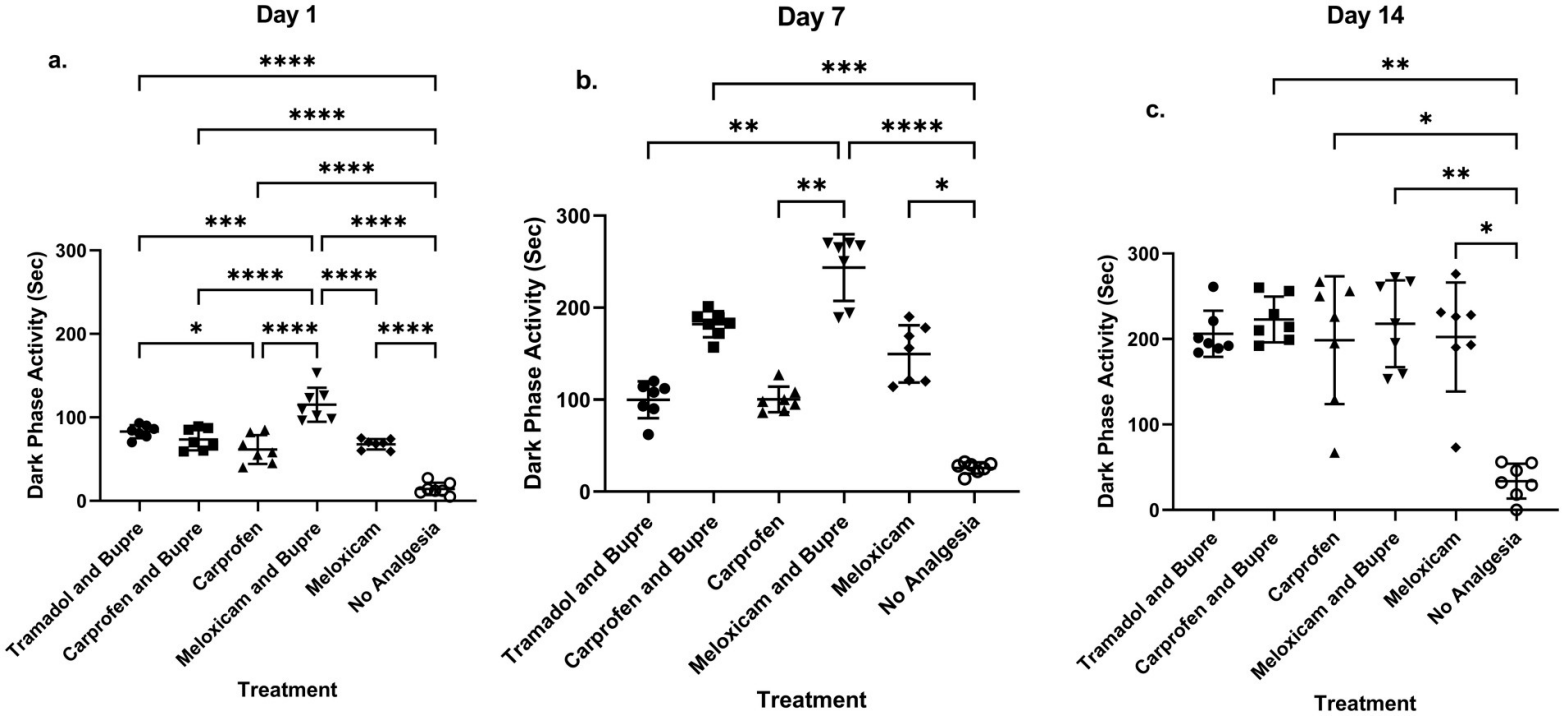
Dark phase activity in home cages showed differences between groups on days 1 (a), 7 (b) and 14 (c). * = P < 0.05, ** = P < 0.01, *** = P < 0.001, **** P <0.0001.

Stereotypic activity was absent in any of the studied groups. Activity observed during the dark phase differed between groups on Day 1 (F (5,36) = 43.73, P = <0.0001), Day 7 (Kruskal-Wallis statistic = 36.85) and Day 14 (Kruskal-Wallis statistic = 17.98) and the activity was lower in the no-analgesia group in comparison to those that received analgesia during all these days (Fig 3).

On *Day 1*, post-hoc analysis revealed that the activity of animals that received no analgesia was significantly lower than in the tramadol and buprenorphine (P<0.0001), carprofen and buprenorphine (P<0.0001), carprofen (P<0.0001), meloxicam and buprenorphine (P<0.0001) and meloxicam (P<0.0001) groups. Activity also differed between groups that received different analgesia on Day 1, where meloxicam and buprenorphine showed higher activity in comparison to other groups viz. tramadol and buprenorphine (P = 0.0007); carprofen and buprenorphine (P<0.0001); carprofen (P<0.0001) and to meloxicam (P<0.0001). Further, tramadol and buprenorphine showed better activity in comparison to carprofen (P = 0.047) as well (Fig 3a).

On *Day 7*, post-hoc analysis showed that the activity of animals that received no analgesia was significantly lower than in the carprofen and buprenorphine (P = 0.0005), meloxicam and buprenorphine (P<0.0001) and meloxicam (P<0.0144) groups. Meloxicam and buprenorphine showed higher activity in comparison to other groups viz. tramadol and buprenorphine (P = 0.0062) carprofen (P = 0.0041) (Fig 3b).

### Days 14

Post-hoc analysis showed that on *Day 14*, the activity of animals that received no analgesia was significantly lower when compared to carprofen and buprenorphine (P = 0.0068), carprofen (P = 0.0228), meloxicam and buprenorphine (P = 0079) and meloxicam (P = 0.0211) groups (Fig 3c).

Activity levels did not differ at the remaining time points between any of the groups.

### Basso beattie bresnahan (BBB) scoring

**Fig 4:**
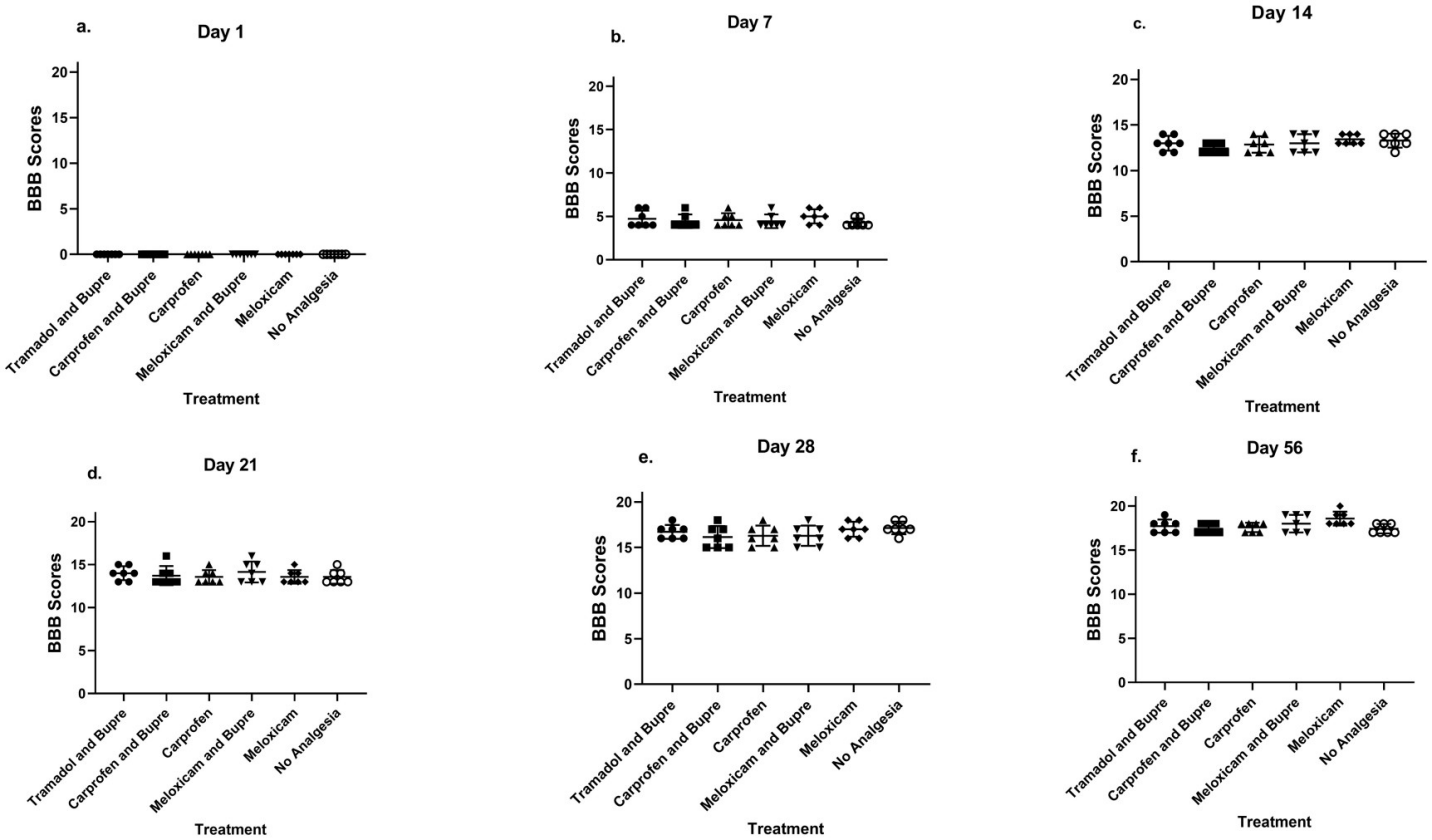
Basso beattie and bresnahan score (BBB). There existed no differences between groups on days 1 (a), 7 (b), 14 (c), 21 (d), 28 (e), and 56 (f) in the BBB scores which is a measure of motor activity functionality in spinal cord injured rats.

BBB scores were zero on the first post-operative day for all the rats in all the groups. The rats in all the groups progressively gained better scores over time averaging 4.6±0.7 on Day 7, 13±0.8 on Day 14, 13.8±1 on Day 21, 16.6±1 on Day 28 and 17.8±0.8 on Day 56. However, none of the groups differed significantly between each other at any of the time points (Fig 4).

### Novel object recognition (NOR) test

**Fig 5:**
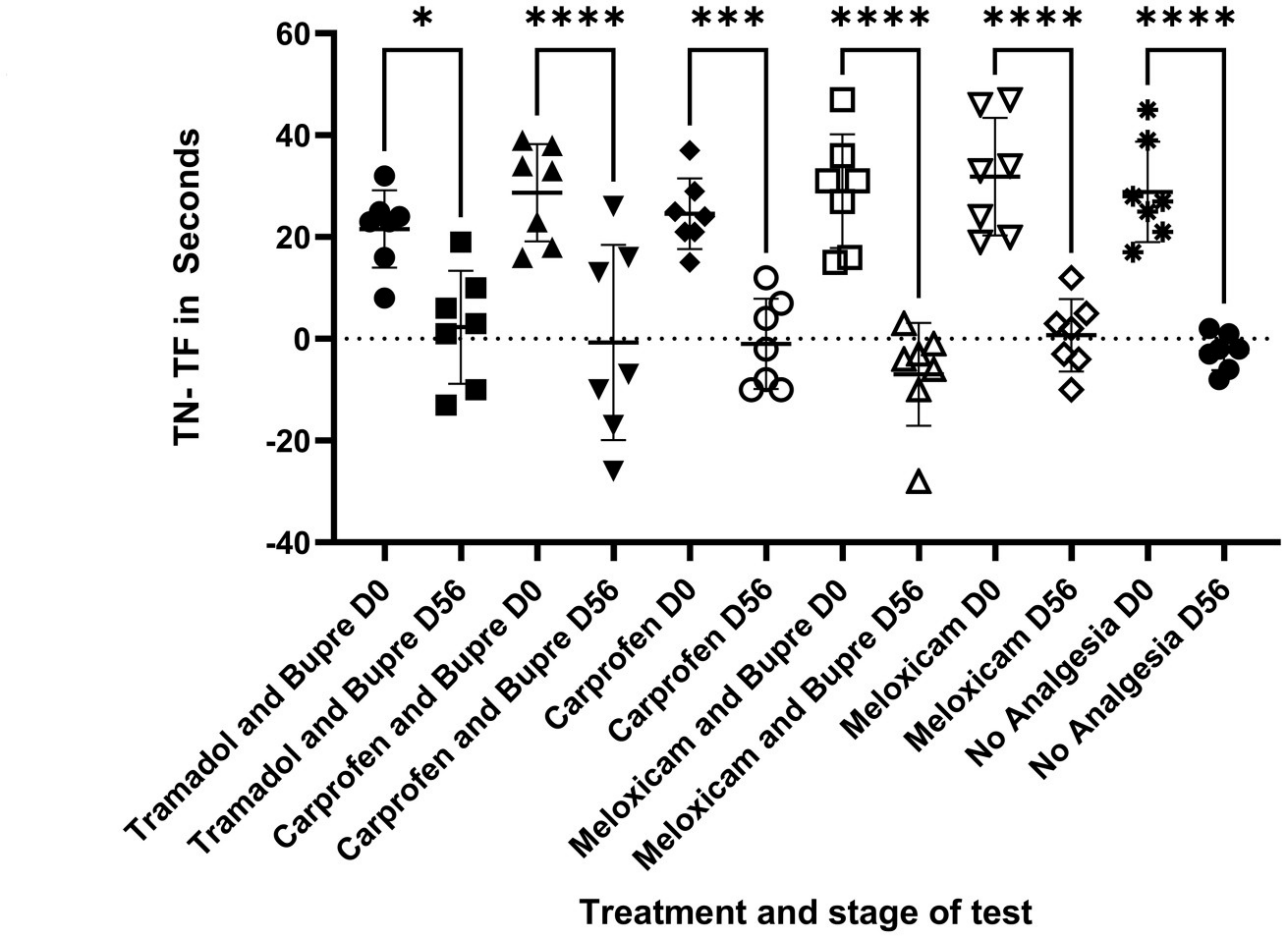
Novel object recognition test (NOR). The time spent (in seconds) to explore the novel object (T_N_) in comparison to familiarised object (T_F_) differed in all the groups before surgery at (Day 0) compared to the end of the study (Day 56). * = P < 0.05, **** P <0.0001.

The time spent to explore the novel object (T_N_) in comparison to familiarised object (T_F_) differed in all the groups before surgery at (Day 0) compared to the end of the study (Day 56). All the groups spent more time with novel object for a significantly higher period when the baseline was obtained (Fig 5). Discrimination Index (DI) did not differ between groups on Day 56 in the NOR test at 5 minutes or at 24 hours. In the 5-minute delayed test, the DI scores measured 0.04±0.18 for the tramadol and buprenorphine group, 0.03±0.35 for carprofen and buprenorphine group, -0.4± 0.14 for carprofen, -0.1± 0.15 for meloxicam and buprenorphine, 0.02± 0.2 for meloxicam group, and -0.04±0.06 for no analgesia group and for the 24 hours delayed test, the DI scores were 0.01±0.09, -0.05±0.2, -0.02±0.2, 0.01±0.2, 0.1±0.2, and -0.1±0.3 respectively.

### von Frey test

Paw withdrawal threshold (PWT) did not differ between groups at the end of the study on Day 56 for both right and left hindlimbs. The PWT scores measured 7.6±3.7 for the tramadol and buprenorphine group, 8.1±3.4 for carprofen and buprenorphine, 8.4± 3.3 for carprofen, 7.3±4 for meloxicam and buprenorphine, 9.0± 3.4 for meloxicam, and 7.3±3.6 for the no analgesia group in the right hind limb. For the left hind limbs, the PWT scores measured 6.8±2.18 for the tramadol and buprenorphine group, 7.0±3.8 for carprofen and buprenorphine, 7.3± 3.6 for carprofen, 7.6±4 for meloxicam and buprenorphine, 7.8± 3.7 for meloxicam, and 8.8±4.4 for the no analgesia group.

### Histopathology

**Fig 6:**
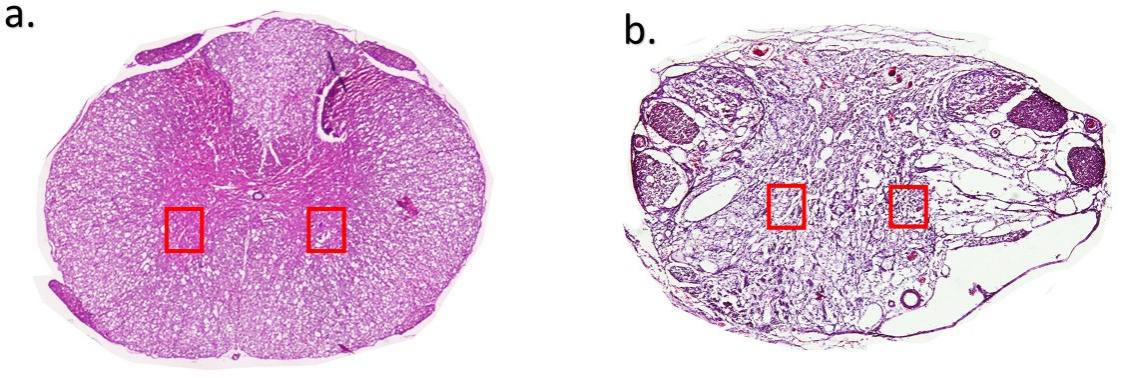
a. Cross section of the spinal cord an intact spinal cord with inset showing the site of analysis, the ventral horn region bilaterally, performed for the entire study. b. Cross section of the spinal cord with spinal cord injury at lesion epicentre with inset showing the site of analysis for area of vacuolation and live neuronal count.

**Fig 7:**
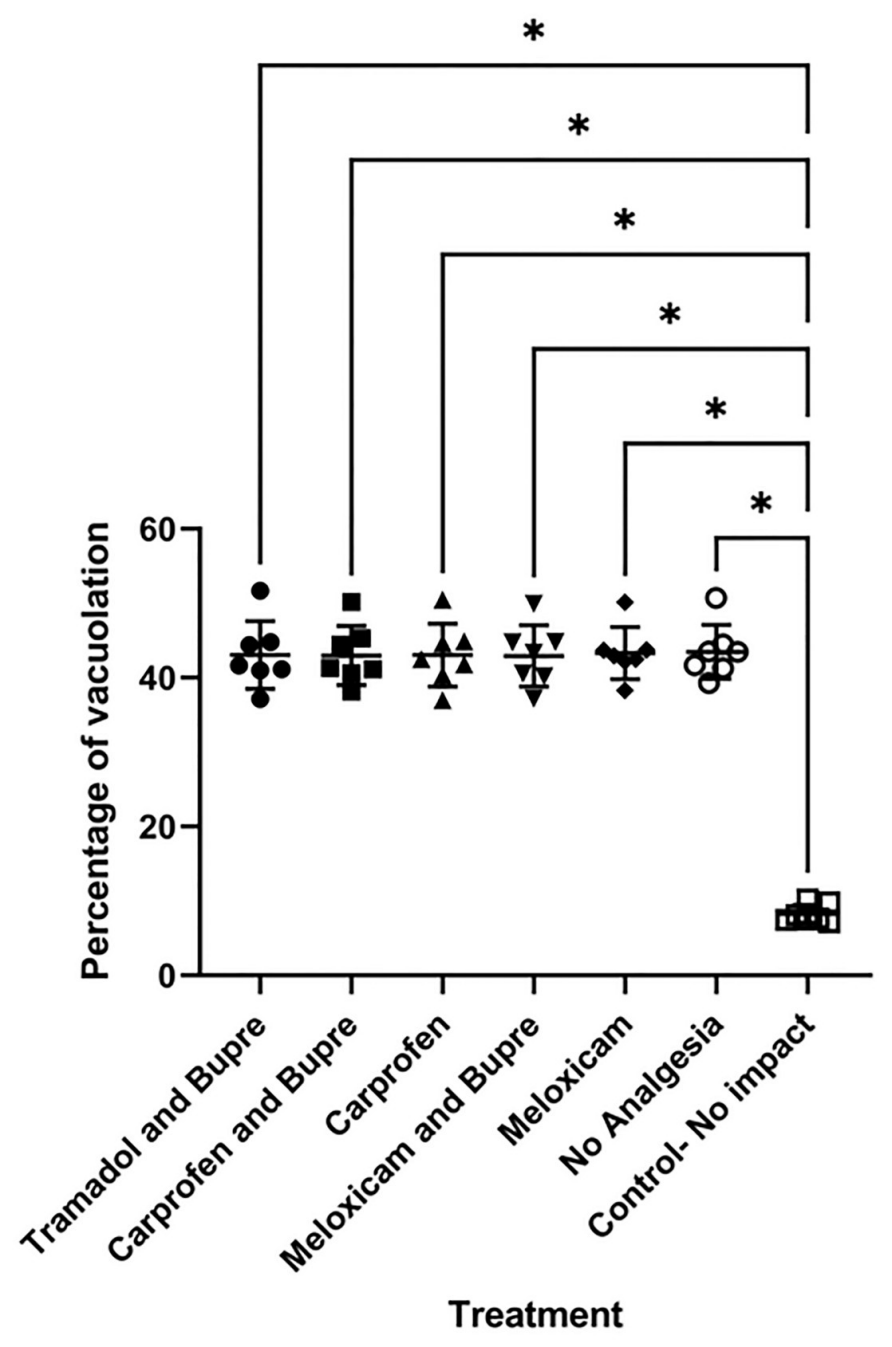
Percentage of vacuolation in ventral horns of spinal cord in rats. All the groups differed significantly in vacuolation area in comparison to the non-SCI control group. * = P < 0.05.

**Fig 8:**
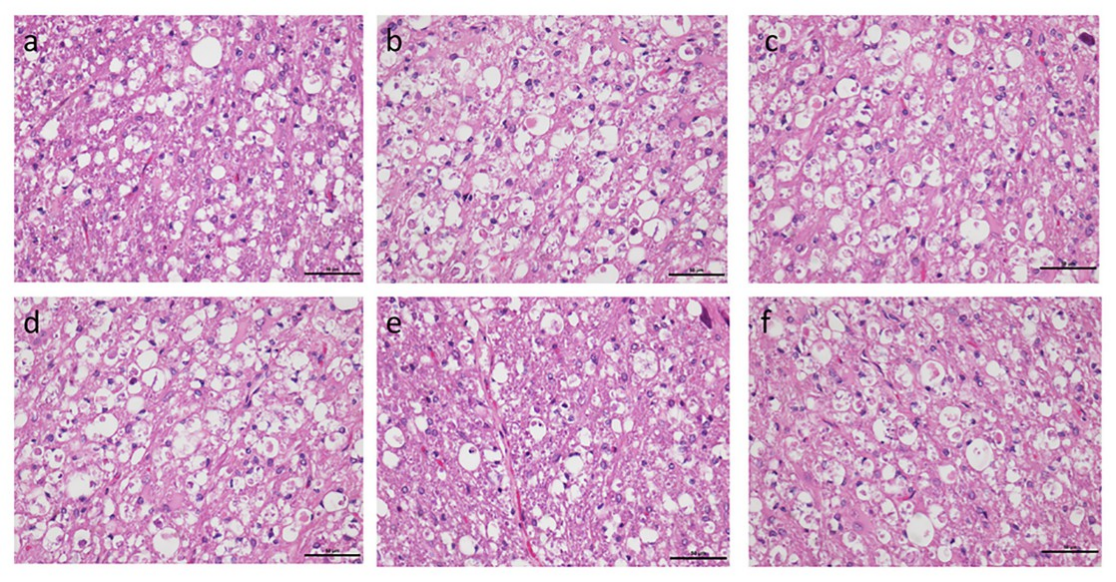
Vacuolation in ventral horns in Hematoxylin and Eosin staining of spinal cord in rats. Representative photographs from each group as analysed for comparison (Fig-8a to 8f represents Groups I to VI respectively, serially in order). The scale-bar used in the picture is 50μm.

The areas considered for analysis at the lesion epicentre from SCI animals are shown as insets (Fig 6a) and the normal architecture of the intact spinal cord as reference is also shown (Fig 6b). Area of vacuolation did not differ between the group that did not receive any analgesia and the groups that received analgesia. The vacuolation area measured in percentage with respect to tissue spared was 43.1±4.4, 43±3.8, 43±4.1, 43±4, 43.3±3.4 and 43.5±3.5 in the tramadol and buprenorphine, carprofen and buprenorphine, carprofen, meloxicam and buprenorphine, meloxicam and no analgesia group respectively. All the groups differed significantly in vacuolation area in comparison to the non-SCI control group in which the percentage of vacuolation measured 8.3**±**12 (Fig 7). Representative photographs from each group as analysed for comparison is shown in Fig 8.

**Fig 9:**
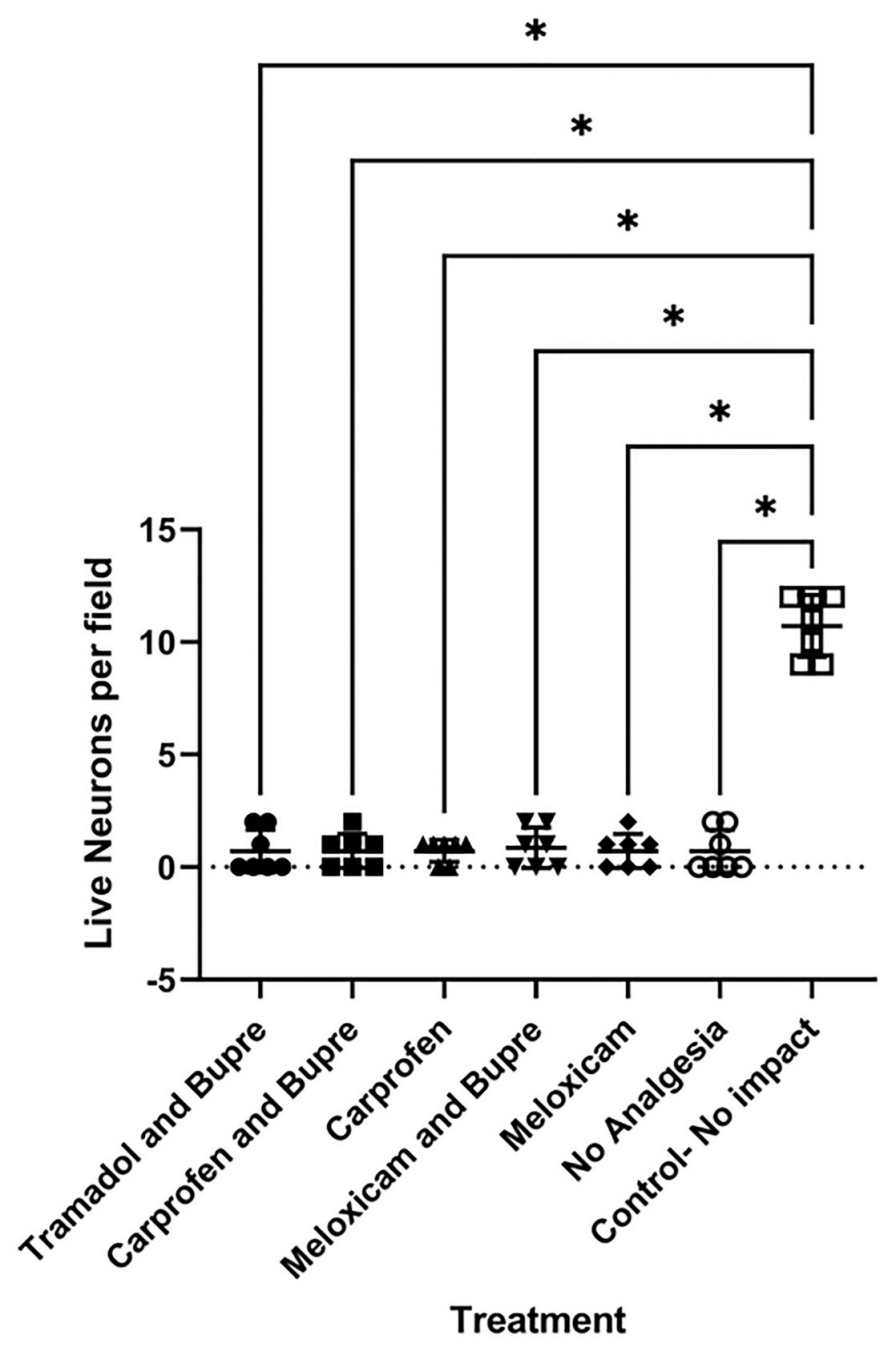
Live neuronal count- All the groups differed significantly in number of live neurons in comparison to the non-SCI control. * = P < 0.05.

**Fig 10:**
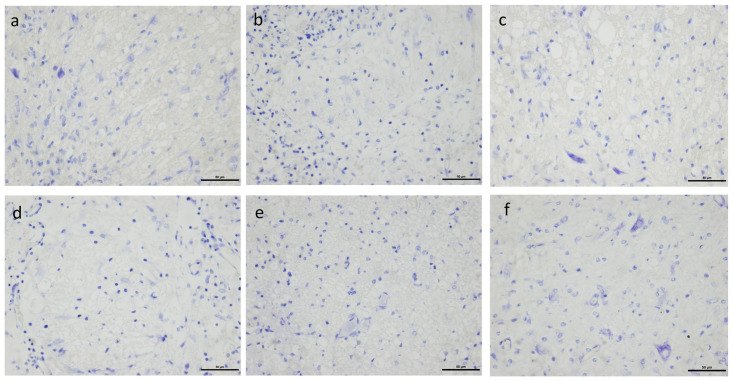
Live neuronal count- Representative pictures of live neurons counted in ventral horns in Nissl’s staining of spinal cord in rats. Live neurons from each group as analysed for comparison is shown (Fig- 10a to 10f represents Groups I to VI respectively, serially in order). The scale-bar used in the picture is 50μm.

Number of live neurons did not differ between the groups that did not receive any analgesia and the groups that received analgesia. Tissue damage was qualitatively assessed and was observed to be of severe. Neurons were scarce in the fields. In the intact control, neurons were visualised in abundance. All the groups differed significantly in number of neurons in comparison to the non-SCI control (Fig 9). Representative pictures from each group as analysed for comparison is shown in Fig 10.

## Discussion

Spinal cord injury is projected as a global health priority owing to its high incidence rates spread across all the continents [33]. The incidence rates have been notably increasing since the past 30 years [34] and the disease severely affects the quality of life by exerting both physical and psychological dysfunctions. The rats are primarily the animal species used as model to study disease progression of the injury and to evaluate therapeutic regimen that are aimed to alleviate the SCI [35, 36]. The use of rats as an SCI model has led several treatment modalities from pre-clinical studies to clinical phase trials [35]. Even though SCI occurring at the cervical level accounts for most of the human cases [37], thoracic SCI remains to be the mostly used area in preclinical studies [38]. This is because of the high mortality in SCI at the cervical level [39]. The improvisations on laminectomy in terms of refinement would have been more effective if hemilaminectomies were performed. However, for the type of work that is performed by us, laminectomies were essential. Even though more than 80% of the incidence rates of SCI occurs in male human population [40], female animals are mostly used for research owing to practical reasons [41]. SCI research is mostly performed using female rats [41–44] owing to the complications arising due to urine retention in paraplegic male rats. On the contrary, it has also been shown that female rats exhibit a faster hind-limb functional recovery post SCI that could be attributable to female sex hormones [45]. Hence, the use of male rats for pre-clinical SCI research should be considered, and studies to investigate possible sex-related difference are currently being planned by our group.

Since there are much studies providing antibiotic prophylaxis [46], our study also followed the same procedure. It shall also be noted that no mortalities or infections occurred in our study. However, the use of antibiotics is indeed a concern, due to the risk of pathogens developing resistance to antibiotic agent. Refraining from the use of antibiotics could be implemented, by careful urine evacuation, but this has to be done with caution and data shall be published based on subsequent findings on the same. In animal experimentation for SCI studies, the use of post-operative analgesics appears to be highly uncommon [47–50]. Further, studies that do report usage of analgesia often provide minimal details on duration of therapy [51] and frequency of administration, or report non-validated analgesic regimens [52] that could be inadequate [53, 54]. Further, several systematic reviews done on animal models of SCI has also overlooked the importance of usage, reporting, and risk of potential side-effects of analgesics on research data, if any [38, 43, 55]. In a previous study, acute SCI was studied for a 14-day time period using BBB score and RGS alone, and it was found that tramadol did not influence the functional recovery in rats [56]. Buprenorphine has also been proven a drug that can be used in SCI without affecting anatomical, physiological or behavioural parameters in rats [57]. Studies are rare to prove that the usage of analgesics can bring in refinement by alleviating unnecessary and lasting pain to the animals used for SCI research.

SCI research is done using rats as the model in over 72% of the studies [38] owing to the closeness of pathogenesis with respect to humans. Most of the researchers either doesn’t use analgesics or report them during the post-operative period in SCI research [58–60].

However, working groups have started to realize the importance of engaging analgesia for SCI studies and to give recommendations to alleviate pain and suffering in these models [61]. Standardisation of analgesic protocols and optimisation of effective combinations of analgesic drugs is an emerging need to enable better comparison of studies, which has been the focus of our study. The present study of ours aimed to add more knowledge about the importance of analgesia and about reporting the same while publishing SCI research using animal models.

### Body weight

It is a well-established fact that, after surgical procedures change in body weight serves as an efficient index to assess post-operative pain, stress or other causes of impaired welfare in experimental rats [62]. The no-analgesia group consistently had lower body weights on Days 7, 14 and 28, in comparison to all the other groups that received analgesia; the only exceptions being on Day 28 where the tramadol and buprenorphine combination as well as the meloxicam and buprenorphine combination did not differ from the no-analgesia group. It has previously been demonstrated that buprenorphine can affect body weight gain in rats in a negative manner [62, 63]. Tramadol has been shown to reduce water intake and could negatively affect animal well-being in certain doses [64]. The appropriate frequency of administration of tramadol to experimental animals remains uncertain [65] and is useful only in procedures with mild to moderate pain for up to 2 h [66]. Hence, these factors could contribute to the lower comparative weights observed in the tramadol and buprenorphine group throughout the study period. Nevertheless, the no-analgesia group remained distinctively affected in terms of body weight in comparison to the groups that received analgesia. Other studies have established that certain analgesics like meloxicam improves, while ketoprofen reduces feed intake and body weight gain [18]. Meloxicam increased the body weight gain as per our study as well. However, carprofen that belongs to the same class of propionic acid NSAIDs as ketoprofen did not produce weight loss in the rats in our study. Our data thus demonstrate that an array of analgesics can be used in post-operative pain management in SCI studies to improve the body weight gain and thereby improve post-operative recovery and well-being. Owing to practical reasons in managing an even bigger group of experimental rats simultaneously, all animals requiring individual daily-multiple time care and data acquisition, tramadol alone and buprenorphine-alone groups were not included in the current work. Moreover, owing to the same reason, we were not able to include more analgesics in the list of drugs studied. It is suggested that the effectiveness and side-effects of any of these drugs as stand-alone analgesic treatment groups shall be tested in future to gather data.

### RGS score

After cervical SCI, it has been demonstrated that RGS can be used to assess spontaneous pain and evoked supraspinal pain sensation in rats, where significantly higher RGS scores were reported after five weeks of SCI [67]. The present study of ours also employed RGS as a tool to detect spontaneous pain from the SCI procedure. However, except for on Day 1 and 7, there were no differences between the groups. The results obtained from the present study clearly demonstrates the ability of the test to differentiate between groups by showing significantly high pain scales in the group that did not receive analgesics. A review paper on pain assessment methods, enlists several chronic pain models such as colitis, cervical radiculopathy, neuralgia, SCI, orofacial pain and migraine in rats and mice in which grimace scales can be used to re-evaluate the doses of analgesic drugs [18]. In contrast, the results from our study could not obtain significant results after Day 7, indicative of the inability of RGS to extract data from chronic pain under the circumstances. This is in agreement with previous findings populated in the review that grimace scales could not find differences between two well characterised chronic pain models in mice [18]. Our results are also consistent with previous results from our research group, where it was shown that RGS could detect significant differences on Days 1 and 7 while two different techniques to produce laminectomy were compared [25]. Hence, RGS appears to be useful for assessing pain for up to about a week post-operatively in the SCI-model, but data regarding assessment of spontaneous pain beyond seems to be inconclusive.

### Dark-phase home cage activity score

Non pain evoked behavioural tests can be used effectively to assess pain and well-being in rodents [18, 68]. Among such tests, monitoring home cage activity is considered to be advantageous over tests like open field and elevated plus maze to observe rodent behaviours, since the home cage provides a stress-free environment allowing the activity at the natural pace of the animals [69]. It is noteworthy that the group that received no analgesics exhibited less activity until Day 14 as evident from data obtained using night vision camera during the dark phase. Previous studies in rats have shown that tramadol has no impact on activity levels, whereas carprofen has been demonstrated to increase the same. In our study, it was demonstrated that tramadol in combination with buprenorphine increased activity on Day 1, which is in contrast with the findings from several other studies reviewed in Foley *et al* [65]. It is therefore likely that the increase observed in the tramadol and buprenorphine group is related to buprenorphine and not to tramadol, since buprenorphine has been shown to increase activity under some circumstances [70]. The results from carprofen, on the other hand, were in agreement with the studies described by Foley *et al* [65], where the activity levels were higher in comparison with the group that received no analgesic treatment. Stereotypic activity screening provides important information on animal wellbeing [71]. In our study, no stereotypic behaviour was observed in any of the groups during the dark phase behavioural analysis. In summary, the present study presents data that supports the hypothesis of improved wellbeing of the animals when administered with post-operative analgesia in terms of home cage dark phase activity.

### BBB Scoring

BBB scoring to assess motor recovery has been the first choice in the majority of SCI models [72]. Since this scoring is highly indicative of the development of the SCI, it was considered important to study this parameter and compare with different types of analgesia applied, to investigate if the analgesic treatment would have any undesired impact on the functionality of the model. Variability in results between studies and within the studies is a concern in SCI research [73]. Recently, high variability observed in BBB scores in rats even after using a force-defined commercially available equipment was reported [74]. The recovery as reported by a severe compression injury using 50g weight at Th9 was of BBB score 10–11, whereas it wasn’t much different for the less-severe weight groups, of 30g and 40g which was 8–9 and were not statistically different [75]. Our animals which were impacted using a custom-made calibrated spring-loaded force deliverer at T11 vertebra, showed a faster recovery in comparison as documented previously at T10 using a novel- force deliverer by Scheff et al. [76]. It is of importance for researchers to note these variabilities being caused by different techniques, as well as the variability in results even when standardised equipment is used. Nevertheless, since the system was handled in a uniform manner in our study, we consider that the comparison of results between groups are valid.

It was noted that no groups differed in the functionality outcome assessed using BBB scores during any of the time points studied. There us thus no reason for refraining the use of adequate analgesia in SCI studies based on this parameter.

### NOR test

Cognitive impairment is a characteristic finding associated with SCI [77] and has been previously demonstrated at 8 weeks after SCI in rats using the Morris Water Maze [78]. The results obtained in our present study also indicates the fact that significant memory loss can occur owing to contusion SCI in rats. Administration of analgesia had no effect in this functional loss of memory, which supports other findings in the present study, that there is apparently little risk with providing the animals with analgesia postoperatively in the SCI model. Dorsal von Frey test results weren’t significantly different between groups that received analgesia in comparison with the group that received no analgesics, similar to what was observed with the other parameters of functionality of the model like NOR and BBB studied in this work. Recently it has been shown that some drugs like mirogabalin [79], naringenin [80], ambroxol [81], can exert long lasting analgesic effects in the SCI rat model by altering paw withdrawal thresholds during the von Frey test. If routinely used analgesics would exert a similar effect, it could affect the desired functional outcome of the model itself and hence impair the validity of the model. However, from the findings in the present study, we could not find any adverse effects exerted by analgesic drugs on the functional outcomes as desired and expected from the model.

### von Frey test

The study employed a consistent use of dorsal von Frey test throughout the study. The motive was to perform the same technique, and since there was a dragging movement exhibited by rats for the first few weeks, placing the animal on a grid to perform the plantar test was considered stressful and thus avoided. Further, the plantar test is more time consuming and is thus stressful to the rats that have already undergone a stressful procedure and are physically challenged. Dorsal testing was considered faster, more effective and holds good for all types of SCI studies in rats.

Dorsal von Frey test results weren’t significantly different between groups that received analgesia in comparison with the group that received no analgesics, similar to what was observed with the other parameters of functionality of the model like NOR and BBB studied in this work. Recently it has been shown that some drugs like mirogabalin [79], naringenin [82], ambroxol [81], can exert long lasting analgesic effects in the SCI rat model by altering paw withdrawal thresholds during the von Frey test. If routinely used analgesics would exert a similar effect, it could affect the desired functional outcome of the model itself and hence impair the validity of the model. However, from the findings in the present study, we could not find any adverse effects exerted by analgesic drugs on the functional outcomes as desired and expected from the model. The time points at which von Frey tests were performed was selected to gather data on acute and chronic pain, and was in accordance with previous studies [25, 83].

### Histopathology

Most of behavioural testing involves a portion of subjectiveness [68]. The risk of observer bias from this fact can be prevented by proper blinding of the observers, which was also the case in the present study. Nevertheless, it is important to also include objective parameters to assess the functionality of the model. Cavities in the form of vacuoles are formed in the rats at the site of spinal injury which is species-specific and thus providing high construct validity to the model [84]. Hence, the assessment of vacuolation area using hematoxylin eosin staining can provide quantitative data to assess functional outcome and to enable fair comparisons between groups. Neuronal cell density assessment using healthy motor neuron counting of area of SCI stained with Nissl’s stain is another dependable technique that is widely used to assess and compare functional recovery [31, 32].

This study confirms that the analgesics used, either as single treatment or in combinations for a multimodal approach, do not affect the functional recovery or outcome of the rat SCI model, as determined from the finding of vacuolation area as well as live neuronal count.

## Conclusion

Our study used a modified technique to perform laminectomy, using a motorised dental burr to mechanise the process to refine the process in terms of animal wellbeing, as previously described [25, 85]. Even though refined techniques to mechanise laminectomy and reduce bleeding, inflammation and trauma has been adopted by certain researchers [86, 87], a majority of publications on the induction of SCI applies the manual, conventional technique to perform laminectomy. So, it is required to replicate this study by performing laminectomy manually to study the effects of analgesia which is also being planned. It is emphasised that, analgesic treatment is a refinement of the model, resulting in better animal welfare in SCI studies, while the intended outcome of the model is not influenced by the various types and combinations of analgesic drugs investigated in the present study.
