# Correction: Refinement of the motorised laminectomy-assisted rat spinal cord injury model by analgesic treatment

**DOI:** 10.1371/journal.pone.0360496

**Published:** 2026-10-13

**Authors:** Harikrishnan Vijayakumar Sreelatha, Hamza Palekkodan, Ansar Fasaludeen, Lissy K. Krishnan, Klas S. P. Abelson

Fig 8 in [1] is incorrect. Panels b-d in Fig 8 erroneously show images from the same slide. Additionally, instead of representing all 6 treatment groups, panels a and e in Fig 8 are images from treatment Group I, and panels b, c, d, and f are images from treatment Group II. A corrected Fig 8 is provided with this notice where panels c-f are replaced so that each panel in Fig 8 represents a different treatment group as stated in the figure caption. The quantitative results reported in Fig 7 are not affected by these errors.

Histopathology images from the original stained tissue slides used for the quantification reported in Fig 7 are on figshare [2].

The authors apologize for the error in the published article.

**Fig 8:**
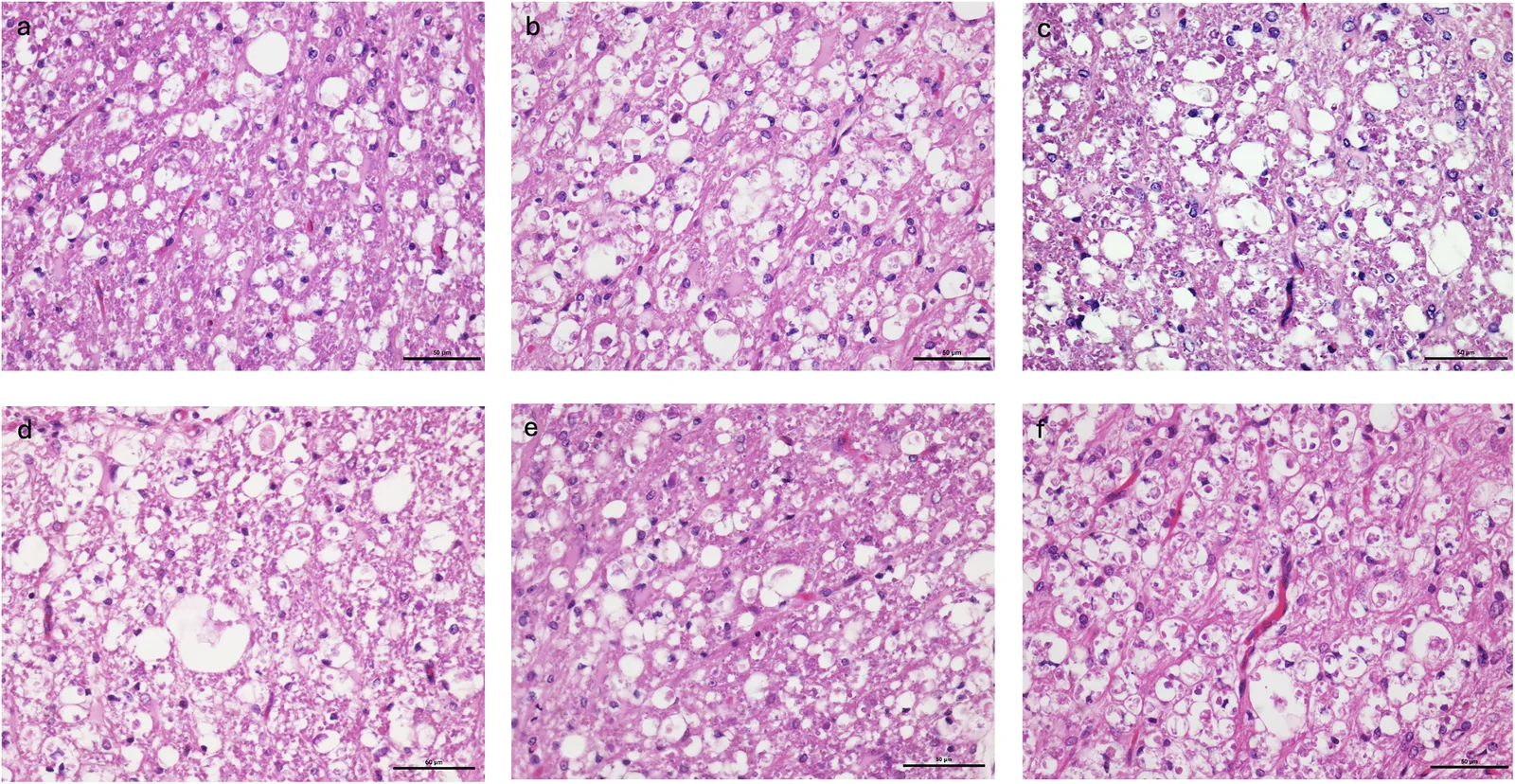
Vacuolation in ventral horns in Hematoxylin and Eosin staining of spinal cord in rats. Representative photographs from each group as analysed for comparison (Fig-8a to 8f represents Groups I to VI respectively, serially in order). The scale-bar used in the picture is 50μm.
